# Cross-Sectional Study of Soil-Transmitted Helminthiases in Black Belt Region of Alabama, USA

**DOI:** 10.3201/eid2912.230751

**Published:** 2023-12

**Authors:** Claudette Poole, Troy Barker, Richard Bradbury, Drew Capone, Amy Hutson Chatham, Sukwan Handali, Eduardo Rodriguez, Yvonne Qvarnstrom, Joe Brown

**Affiliations:** University of Alabama at Birmingham, Birmingham, Alabama, USA (C. Poole, A. Hutson Chatham);; University of North Carolina, Chapel Hill, North Carolina, USA (T. Barker, J. Brown);; Federation University, Australia (R. Bradbury);; Indiana University, Bloomington, Indiana, USA (D. Capone);; Centers for Disease Control and Prevention, Atlanta, Georgia, USA (S. Handali, E. Rodriguez, Y. Qvarnstrom)

**Keywords:** helminths, parasites, soil-transmitted helminths, hookworm, children, Black Belt, Alabama, United States

## Abstract

We conducted a cross-sectional study to determine the prevalence of soil-transmitted helminthiases (STH) in areas of rural Alabama, USA, that have sanitation deficits. We enrolled 777 children; 704 submitted stool specimens and 227 a dried blood spot sample. We microscopically examined stool specimens from all 704 children by using Mini-FLOTAC for helminth eggs. We tested a subset by using molecular techniques: real-time PCR analysis for 5 STH species, TaqMan Array Cards for enteric helminths, and digital PCR for *Necator americanus* hookworm. We analyzed dried blood spots for *Strongyloides stercoralis* and *Toxocara* spp. roundworms by using serologic testing. Despite 12% of our cohort reporting living in homes that directly discharge untreated domestic wastewater, stool testing for STH was negative; however, 5% of dried blood spots were positive for *Toxocara* spp. roundworms. Survey data suggests substantial numbers of children in this region may be exposed to raw sewage, which is itself a major public health concern.

Safe water and sanitation are fundamental to public health (*1*,*2*). Breakdowns in those systems lead to disease and, in temperate and tropical climates, soil-transmitted helminthiases (STH). STH are parasitic infections adversely affecting health, particularly in children and pregnant women, by contributing to anemia and malnutrition (*3*). An estimated 1 billion persons are infected with STHs worldwide, largely in low- and middle-income countries (*3*). Although the wastewater infrastructure need in such countries is well-reported, underserved communities in the United States may also lack basic services, including effective sanitation (*4*–*6*). In 2011, the United Nations’ special rapporteur on the human right to safe drinking water and sanitation reported many failures in the United States (*7*), including in Alabama’s Black Belt region (*8*), where many households lack effective wastewater systems (*9*,*10*). The Black Belt region, named for its rich black soils, is characterized by extreme poverty, poor health outcomes, limited healthcare access, limited economic opportunities, and other challenges (*11*,*12*).

*Necator americanus* hookworms were prevalent in Alabama until the mid-1900s (*13*). In 1929, the highest prevalence (26%–75%) was observed in coastal counties with sandy soils, whereas counties in northern Alabama had much lower prevalence (1%–5%). A moderate prevalence was noted in the Black Belt counties (Lowndes [24%], Wilcox [44%], and Perry [45%]) (*14*). Large-scale public health efforts, supported by the Rockefeller Sanitary Commission and the state and local health departments, attempted to eradicate hookworm. A survey of 13 Alabama counties in 1937, and again in 1951, showed a decrease in prevalence from 37% to 17% among school age children (*15*). However, few systematically collected data have been available since the 1950s.

In the early 1990s, rural healthcare providers in the Alabama Black Belt continued to empirically treat children for STH, but microscopically confirmed cases of hookworm were not reported (*16*). Subsequently, hookworm in Alabama received limited attention until a study published in 2017, reported an analysis of 55 positive stool samples in which 19 (35%) were positive by qPCR for *N. americanus* hookworms and 4 (7.2%) were positive for *Strongyloides stercoralis* roundworms, from a cohort of mostly adults living with poor sanitation conditions in Lowndes County (*17*). Those results, combined with reports of widespread wastewater sanitation failures, raised the possibility of continued STH transmission in the southeastern United States. Our study objective was to estimate the prevalence of STH among children in rural Alabama.

## Methods

### Study Design and Setting

We calculated sample size by using a prevalence range of 3%–30% on the basis of recent published reports (*16*,*17*). By using an estimated prevalence of 3% (the theoretically lowest prevalence to support ongoing transmission) (*18*,*19*) with an infinite population size and a precision of 1.5%, we determined that a sample size of 497 was needed, giving 95% binomial exact CIs of 1.7%–4.9% with 15 observed events.

We selected 3 counties in the Alabama Black Belt as the study site because of previously reported STHs in Lowndes County (*17*), community concerns regarding water and sanitation in Wilcox County (*20*), and the longstanding failure of the sewer treatment facility in Perry County (*21*). We invited residents of those counties to enroll their children through several recruitment strategies, including word-of-mouth by trusted community leaders, flyer distribution, and advertisements in local newspapers, social media, and radio. Any child 2–18 years of age who had resided for >1 year within the study region were eligible to enroll; however, we used community partners to help identify households most at risk on the basis of levels of poverty, known housing clusters without functioning sanitation, or living close to the failing sewer facility. Enrollment occurred during December 2019–August 2022.

### Survey

We obtained informed consent from guardians and assent of children >7 years of age and administered a short paper survey. We collected demographic data, contact information, and preference for treatment by the project physician or personal physician (if an infection was found). The survey asked about possible risk factors for infections, including household sanitation type, home sewage contamination, domestic animal exposure, well-water consumption, home-grown produce consumption, international travel history, and exposure-limiting behaviors such as screen time. The survey also assessed prior treatment for STH.

### Sample Collection

During December 12, 2019–March 31, 2020, we obtained finger-prick blood samples on dried blood spot cards (PerkinElmer, https://www.perkinelmer.com) that were shipped to the Centers for Disease Control and Prevention (CDC), Center for Global Health, Division of Parasitic Diseases and Malaria, for multiplex serologic antibody detection for *Strongyloides stercoralis* and *Toxocara* spp. (*22*). We gave families at-home stool collection kits and asked them to deliver self-collected stool specimens for shipment to the University of North Carolina Chapel Hill (UNC) for analysis. During April 1, 2020–August 10, 2022, because of the COVID-19 pandemic, we stopped collecting finger-prick blood samples and asked participants to mail the self-collected stool specimens directly to the UNC laboratory in prepaid packaging. We asked participants to collect stools from 3 separate bowel movements on separate days, then fill two 50-mL collection tubes each with 15 g of stool (1 containing 15 mL of 10% formalin and another containing 15 mL of zinc polyvinyl alcohol [Zn-PVA] [Parapak; Meridian Bioscience, https://www.meridianbioscience.com]). This method enabled preservation of stool specimens at ambient temperature for transportation to the laboratory. We offered participants monetary stipends on receipt of adequate stool specimens ($25 for the first specimen, $50 for the second, and $75 for the third).

### Microscopic Analysis

Upon receiving the specimens at the UNC laboratory, we homogenized the specimens by using sterile inoculating loops (VWR, https://us.vwr.com). We stored formalin-preserved stools at ambient temperature and stored Zn-PVA–preserved stools at 4°C. Trained laboratory technicians used the mini-FLOTAC method (*23*) to identify and enumerate helminth eggs from formalin-preserved samples (Appendix). In brief, we homogenized 4 grams of the stool-formalin mixture with 36 mL of sodium nitrate (VWR) solution (specific gravity 1.25) in a fill-FLOTAC and then dispensed them into 3 mini-FLOTAC disks. After 10 minutes, we turned and read the disks at 100× magnification by using a trinocular light microscope (VWR). The theoretical limit of detection of this method was 3.3 eggs/g (*24*). We photographed suspected eggs by using a mounted camera (Motic, https://www.motic.com) and sent images to CDC’s DPDx telediagnosis service (https://www.cdc.gov/dpdx/index.html) for morphologic confirmation.

### Molecular Analysis by TaqMan Array Card and Digital PCR

After homogenization, we extracted nucleic acids from 150 mg of selected Zn-PVA preserved stool by using the QIAamp 96 Virus QIAcube HT Kit (QIAGEN, https://www.qiagen.com), which included a pretreatment step using Precellys SK38 bead beating tubes (Bertin Technologies, https://www.bertin-technologies.com) (*25*–*27*). We typically extracted samples within 1–4 weeks of receipt (median 15 days, interquartile range [IQR] 8–28 days, range 1–405 days); we extracted 92% of samples within 8 weeks. Among children who submitted >1 stool specimen, we randomly selected a single replicate for extraction. We randomly selected ≈5% of stools for duplicate extraction and another 3% for extraction from multiple replicates. We included >1 extraction-negative control (*28*) during each day of extractions and spiked samples with 10^7^ copies of phage MS2 and 10^6^ gene copies of synthetic DNA (IDT, https://www.idtdna.com) as extraction-positive controls. We stored extracts at –80°C until analysis. We assessed extracts from specimens suspected to potentially be from nonhuman sources by using digital PCR (dPCR) (QIAcuity 4; QIAGEN) for human mitochondrial DNA (*29*).

**Figure 1:**
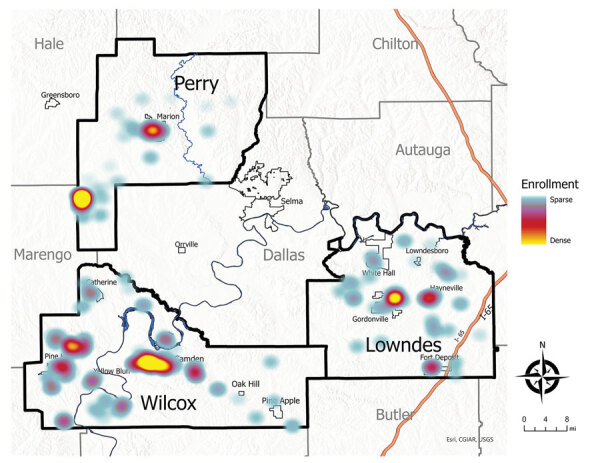
Heat map demonstrating home location distribution of children enrolled in a study of soil-transmitted helminthiases conducted in Lowndes, Wilcox, and Perry Counties, Alabama, USA, December 2019–August 2022.

At the UNC laboratory, we analyzed nucleic acids for 7 helminths by using a custom TaqMan Array Card (TAC) on a Quantstudio 7 Flex (ThermoFisher Scientific, https://www.thermofisher.com), following the methods described in Liu et al. (*30*) The targets included were *Ancylostoma duodenale*, *Ascaris lumbricoides*, *Enterobius vermicularis*, *N. americanus*, *Rodentolepsis (Hymenolepsis) nana*, *S. stercolaris*, and *Trichuris trichiura*. We prepared the TAC by combining 40 µL of template with 60 µL of AgPath-ID One-Step RT-PCR Reagents (ThermoFisher Scientific). We evaluated the TAC performance by using an 8-fold dilution series (10^9^–10^2^ gene copies per reaction) of an engineered combined positive control that was developed using the methods from Kodani and Winchell (*31*). Linearity and efficiency for the six targets were within normative standards (linearity 0.99–1.0, efficiency 95%–100%) (Appendix Tables 1, 2, Figure 1). Each day of TAC analysis, we ran >1 positive and negative (either an extraction-negative control or a PCR-negative control). We determined quantification cycle values by manual thresholding and included comparison of each specimen’s fluorescent signal against the daily negative and positive controls (Appendix Figure 2). We categorized any target that amplified past a quantification cycle of 35 as negative to reduce the potential for false positives (*30*).

**Figure 2:**
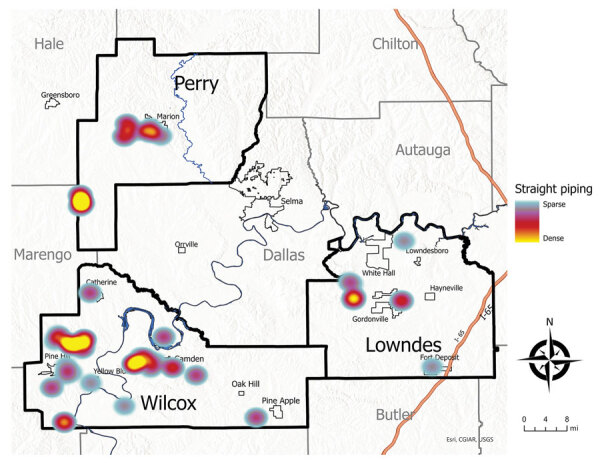
Heat map demonstrating distribution of children enrolled living in homes with self-reported straight pipe sewage discharge in a study of soil-transmitted helminthiases conducted in Lowndes, Wilcox, and Perry Counties, Alabama, USA, December 2019–August 2022.

In addition, we analyzed nucleic acids available from children living in Lowndes and Wilcox counties for *N. americanus* DNA by using dPCR because of its higher sensitivity (Appendix Tables 3, 4, Figure 2). We prepared reactions with QIAcuity Probe Mastermix (QIAGEN) by using 200 nM forward and reverse primers, 800 nM probe, and 4 μL of template. Thermocycling conditions were 95°C for 2 min, followed by 45 cycles of 95°C for 15 s and 55°C for 60 s. We included >1 positive and negative control on each dPCR nanoplate. We set the threshold manually between the bands of the positive and negative controls. We classified specimens with <3 positive partitions as negative (Appendix Table 4).

### Molecular Analysis by Multiparallel Quantitative PCR

We aliquoted 2 mL of Zn-PVA stool samples into sterile cryovials, stored them at 4°C, and shipped them to CDC’s Division of Parasitic Diseases and Malaria for qPCR analysis. We removed the preservative and extracted DNA from 500 mg stool by using either DNeasy PowerSoil Kit or DNeasy PowerSoil Pro Kit (QIAGEN). Eggs were broken up through bead beating in FastPrep-24 homogenizer (MP Biomedicals, https://www.mpbio.com) for 3 min at 6.5 m/s. We performed the DNA extraction procedure in the QIAcube automated nucleic acid purification system (QIAGEN) following the manufacturer’s instructions. We quality control tested DNA extracts for presence of potential amplification inhibitors by using a human cytochromeB gene qPCR (*32*). We tested DNA samples without inhibition by using multiparallel qPCR assays specific *for N. americanus*, *A. duodenale*, *T. trichiura*, *S. stercoralis* (*33*), and *A. lumbricoides* (*34*). We performed qPCR reactions in a total volume of 25 μL, consisting of 250 nM of each primer, 125 nM of probe (Platinum Quantitative PCR SuperMix-UDG w/ROX; ThermoFisher Scientific), and 2 μL of DNA template. Each qPCR run was accompanied by positive (genomic DNA from STH worms) and negative (water and DNA extracted from STH-free feces) amplification controls. We performed the qPCR on an AriaMx Real-Time PCR System (Agilent, https://www.agilent.com) with the following cycling conditions: 50°C for 2 min, 95°C for 2 min, then 40 cycles of 95°C for 15 s and 59°C for 60 s.

### Antibody Detection for *Toxocara* spp. and *S. stercoralis*

We performed detection of antibodies against *Toxocara* spp. and *S. stercoralis* on dried blood spots by using Luminex assay as previously described (*35*,*36*). In brief, we placed the dried blood spots in 0.25 mL of elution buffer at 4°C overnight. We allowed antibodies in the eluate to bind to recombinant antigens *T. canis* C-type lectin and 31 kDa third stage *S. stercoralis* larval antigen coupled to beads. We detected bound antibodies by using R-phycoerythrin reporter (ThermoFisher) in a MAGPIX reader with xPONENT software (ThermoFisher). We considered samples positive at >8 median fluorescence intensity for *S. stercoralis* and 23.1 median fluorescence intensity for *Toxocara* spp. We determined cutoff points by testing sets of defined positive, negative, and cross-reactive serum samples and analyzing the results by receiver operating characteristics curve. We logged in data from case report forms and laboratory results into REDCap (https://www.project-redcap.org) and analyzed the data by using SAS version 9.4 (SAS Institute Inc., https://www.sas.com). We estimated combined sensitivity resulting from multiple microscopic and molecular assays by using surrogate canine hookworm (*Ancylostoma caninum*) (Appendix Table 5, Figures 3, 4).

### Ethics Considerations

This study was approved by the institutional review boards of the University of Alabama at Birmingham (approval no. 300002219), Georgia Institute of Technology (approval no. H19021), and UNC (approval no. 20–3212). The study was reviewed by CDC and conducted consistent with applicable federal laws and policy.

## Results

We enrolled 777 eligible participants from 442 unique households, representing ≈10% of the children living in the study area (Table 1, Table 2; Figure 1). The higher density of enrollment overlapped with higher density of households. Of enrolled children, 93 (12%) reported living in homes with a straight pipe, discharging untreated sewage in the yard or nearby (Figure 2). A total of 227 participants submitted dried blood spot samples, and 704 participants submitted stool samples; 676 children submitted >3 separate stool samples. For 169 participants, we collected both blood and stool samples.

**Table 1:** Characteristics of 777 participants based on self-administered surveys conducted in Lowndes, Wilcox, and Perry Counties, Alabama, USA, December 2019–August 2022*

Characteristic	No. (%)	No. missing
Age, y, mean (SD), median (range)†	10.6 (4.4), 11 (2–18)	7
Years living in current house, mean (SD), median (range)	8.1 (4.8), 8.0 (0–18.0)	18
Sex		
F	393 (50.8)	4
M	380 (49.2)	
Race		
Black or African American	734 (95.2)	6
White	21 (2.7)	
Unknown	2 (0.3)	
Prefer not to answer	14 (1.8)	
Ethnicity		
Hispanic or Latino	11 (1.6)	89
Not Hispanic or Latino	635 (92.3)	
Unknown	6 (0.9)	
Prefer not to answer	36 (5.2)	
County of residence		
Wilcox	352 (45.3)	
Lowndes	132 (17.0)	
Perry	293 (37.7)	
Animals		
Dogs	331 (43.3)	13
Cats	121 (15.8)	
Pigs	7 (0.9)	
None	380 (49.7)	
Other (horse, chicken)	10 (1.3)	
Contact with soil		
Never	233 (30.3)	8
Less than once a month	323 (42.0)	
At least monthly	191 (24.8)	
Not sure	22 (2.9)	
Eat produce from home garden		
Yes	357 (46.7)	13
No	407 (53.3)	
Traveled outside the United States in past 5 y		
Yes	14 (1.8)	13
No	750 (98.2)	
Sewer connection	227 (29.6)	11
Septic tank	312 (40.7)	
Cess pit	2 (0.3)	
Straight-pipe	94 (12.3)	
Don’t know	125 (16.3)	
Other	6 (0.8)	
Sewage contamination of property in the past year		
Yes	62 (8.4)	35
No	680 (91.6)	
If yes, where was the contamination?		
Inside the house	13 (24.5)	9
In the yard	40 (75.5)	
Payment of water bill		
Yes	643 (83.3)	5
No	123 (15.8)	
Don’t know	6 (0.8)	
Amount of screen time daily		
<2 h	128 (16.8)	13
2–4 h	336 (44.0)	
>4 h	300 (39.3)	
Believe screen time prevents child from playing outdoors		
Yes	179 (23.6)	20
No	578 (76.4)	
No. stools received		
0	73 (9.4)	
1	8 (1.0)	
2	20 (2.6)	
3	676 (87.0)	

*Values are no. (%) except as indicated. †Age was calculated on the basis of time between (self-reported) date of birth and date of form completion.

**Table 2:** Percentage of children enrolled, by age group and race per county population, in a study conducted in Lowndes, Wilcox, and Perry Counties, Alabama, USA, December 2019–August 2022*

Characteristic	Lowndes County	Perry County	Wilcox County
Age group, y			
<5	1.5	7.6	2.2
5–9	5.1	13.8	9.8
10–14	9.1	14.8	24.8
15–19	5.5	8.2	14.1
Race			
Black or African American	6.4	12.6	16.5
White	1.6	0.7	1.7

*Based on 2020 US Census data.

### Antibody Detection on Dried Blood Spots

Of the 227 dried blood spots analyzed, 8 were inconclusive because of insufficient sample and 11 tested positive for *Toxocara* antibodies, resulting in a positive exposure rate of 5%. None were positive for *S. stercoralis* antibodies.

### Control Sample Results for Molecular Detection Methods

For PCR run on the TAC platform at the UNC laboratory, the extraction-positive control consistently amplified (median cycle threshold 18), indicating no inhibition present. We observed no contamination among extraction-negative controls (n = 19) or PCR-negative controls (n = 2), and our PCR-positive controls (n = 30) exhibited the expected amplification for all targets (Appendix Table 2). We observed no contamination among any template controls (n = 16) for dPCR, and positive controls exhibited positive partitions (n = 14) (Appendix Table 4). At CDC, 11 DNA extracts (0.6%) showed amplification inhibition and were thus excluded from further testing.

### Microscopic Examination and Molecular Results

We observed no STH eggs through microscopic examination on any stool sample received from the 704 eligible children who submitted stool samples to the UNC laboratory. Aliquots from samples with sufficient volume (1,803 stools from 625 children) were also tested at the CDC by multiparallel qPCR assays specific for *N. americanus*, *A. duodenale*, *T. trichiura*, *S. stercoralis*, and *A. lumbricoides*; all results were negative. We randomly selected a subset of samples for additional testing by 2 different molecular methods at the UNC laboratory; we analyzed 1 stool each from 488 children on TAC and 265 on dPCR. We observed *E. vermicularis* eggs in stool from 2 children (0.28% [2/704]) by microscopic examination and detected *E. vermicularis* DNA in 2 samples (0.41% [2/488]) by TAC. We did not detect DNA from *A. duodenale*, *A. lumbricoides*, *H. nana*, *N. americanus*, *S. stercolaris*, or *T. trichiura* by using the TAC platform, and we did not detect DNA from *N. americanus* by using dPCR.

### Combined Sensitivity

In recovery experiments using canine hookworm (*Ancylostoma caninum*) (Appendix), for 10% formalin at ambient temperature we observed a 0.005 log_10_ reduction in egg count per day; for Zn-PVA at ambient temperature we observed a 0.033 log_10_ in gene copies per day, and at 4°C we observed a 0.015 log_10_ reduction in gene copies per day (Appendix Figures 3, 4). A 2-week gap typically occurred from sample collection to receipt at the laboratory (median 14 days, IQR 11–21 days); we extracted DNA approximately 2 weeks later (median 15 days, IQR 8–28 days), and we usually performed mini-FLOTAC within 2 weeks of receipt (median 13 days, IQR 4–28 days). The 95% limits of detection were 4.0 gene copies/μL template for the *N. americanus* qPCR assay and 0.43 gene copies/μL template for the dPCR assay (Appendix Figure 1). In addition, we estimated that a single undeveloped *A. caninum* ova on average contained 2,220 gene copies of our target sequence (Appendix Figure 4).

Considering this time-dependent reduction in targets (i.e., eggs and DNA), we calculated the estimated sensitivity by assay and the combined sensitivity for a single child shedding 1–100 eggs/g of stool (Table 3). We estimated 100% combined sensitivity to detect hookworm eggs at a concentration of 7 eggs/g (accounting for recovery), which is at the low end of a light infection as defined by the World Health Organization (i.e., 1–1,999 eggs/g) (*37*). We also estimated assay and combined sensitivity without considering recovery to demonstrate the theoretically ideal performance of our methods. Not accounting for recovery, we estimated 100% combined sensitivity at a concentration of 3 eggs/g (Table 3).

**Table 3:** Estimated sensitivity to detect STH infection by assay method in a single infected child for different assumed intensity infections (for fecal testing methods used in a STH prevalence study conducted in Lowndes, Wilcox, and Perry Counties, Alabama, USA, December 2019–August 2022*

Egg/g feces from 1 child	Mini-FLOTAC, triplicate, %	qPCR, single, %	dPCR, single, %	Combined, %
Sensitivity accounting for recovery				
1	2	2	18	20
3	5	6	53	55
5	9	10	89	90
7	12	14	100	100
10	17	20	100	100
100	93	100	100	100
Sensitivity not accounting for recovery				
1	2	10	90	91
3	7	30	100	100
5	12	51	100	100
7	17	71	100	100
10	23	100	100	100
100	99	100	100	100

*dPCR, digital PCR; qPCR, quantitative PCR.

## Discussion

Our survey findings confirmed that a substantial number of homes in our study region lack adequate sanitation, resulting in potential exposure of children to untreated sewage. However, we did not identify any cases of STH, a finding in contrast to McKenna et al. (*17*), who reported 19 cases of *N. americanus* infection and 4 cases of *S. stercoralis* infection among 55 persons in Lowndes County. They detected cases through qPCR at very low concentration by using a standard curve from a previous study, translating to an estimated mean burden of 1–2 eggs/g. Subsequent microscopic examination of specimens from 9 of the 19 positive persons by the Alabama Department of Public Health and the CDC did not detect any hookworm eggs. *Toxocara* seroprevalence was higher in our Alabama cohort than in the national study in comparable age ranges (3.0% in ages 6–11 years, 3.9% in ages 12–19 years) (*38*), indicating higher levels of exposure in the American Southeast, as is also demonstrated in a recent Mississippi surveillance study (*22*). Detection of *E. vermicularis* pinworms in stool samples from our cohort was rare.

There are several factors to consider why our results differ to the McKenna et al. (*17*) study. In the McKenna et al. study, participants were mostly adults and were tested in 2013. In endemic populations, the prevalence of hookworm and *S. stercoralis* threadworm increases with age (*39*,*40*) because adult worms can live in the gut for several years (*41*); thus, although residual infections were reported by McKenna et al., transmission may have since ceased. We only enrolled children because they are most at risk for adverse outcomes associated with STH infection, including anemia (*42*), cognitive deficits, potential growth faltering (*43*), and other outcomes (*44*). In addition, our studies used different methods for sample preservation before analysis; the McKenna et al. study processing stool stored initially on dry ice for up to 5 days, followed by storage until analysis at –20°C (time from collection until analysis not reported).

Conclusive evidence on whether endemic human hookworm exists in rural Alabama would be the identification of a case according to standard diagnostic criteria (observation of >1 definitive hookworm eggs by microscopic examination of a stool specimen), without the possibility of having acquired the infection outside Alabama. To our knowledge, such evidence has not been demonstrated in the recent past. A review of Medicaid claims data from 2010–2018 indicated that STH infections continue to be clinically diagnosed in children in Alabama, but rarely (*45*). Without confirmatory stool diagnostic data, drawing conclusions regarding ongoing transmission is difficult because such diagnoses are frequently made empirically on the basis of parental reports of seeing worms in the stool. In 1991, microscopic examination of stool samples collected from children in Wilcox County identified 3 cases of *A. lumbricoides* infection out of 81 samples collected (*16*). The last published population-based survey using microscopic examination to identify STH eggs in stool samples in the United States found a single positive case of hookworm in a sample of 561 children 3–7 years of age in Kentucky in 1982 (*46*).

Sustained hookworm transmission requires 3 factors: infected persons shedding eggs; environmental conditions for eggs to mature into larvae, typically in sandy soil where temperature and moisture conditions are favorable (*47*); and exposure to susceptible new hosts through contact of the larvae with skin (*47*). In settings with endemic hookworm transmission, studies indicate that some persons within a population shed large numbers of eggs, sufficient to maintain transmission to others, whereas other persons may have moderate- or low-intensity infections (*47*,*48*). If hookworm were endemic to this region, we would expect to have identified some cases with microscopically detectable hookworm eggs. The negative results from microscopic examination were concordant with more sensitive qPCR and dPCR assays we performed on a subset of samples. In addition, we analyzed triplicate samples from 129 persons from Lowndes County, in contrast to McKenna et al. (*17*), who tested single samples from only 55 persons (*48*,*49*). Whereas our survey possibly could have missed isolated infections in this population, we do not consider that result likely given what is known about endemic hookworm transmission. We estimate high combined sensitivity for light infections (<100 eggs/g) in the subset of participants that was tested with all methods.

In conclusion, our study did not confirm endemic STH infection in the Alabama Black Belt. However, our survey data suggests a considerable number of children in this region may be exposed to raw sewage, which is itself a major public health concern. 

Appendix Additional information about cross-sectional study of soil-transmitted helminthiases in Black Belt Region of Alabama, USA.
