# Supplementary material for: Cross-Sectional Study of Soil-Transmitted Helminthiases in Black Belt Region of Alabama, USA
Source: Emerg Infect Dis. 2023 Dec;29(12):2461–70. doi: 10.3201/eid2912.230751 (PMC10683802; doi:10.3201/eid2912.230751)
Supplement: Appendix — Additional information about cross-sectional study of soil-transmitted helminthiases in Black Belt Region of Alabama, USA. [file 23-0751-Techapp-s1.pdf]

Article DOI: https://doi.org/10.3201/eid2912.230751

EID cannot ensure accessibility for supplementary materials supplied by authors. Readers who have difficulty accessing supplementary content should contact the authors for assistance.

Cross-Sectional Study of Soil-Transmitted Helminthiases in Black Belt Region of Alabama, USA

Appendix

Microscopy Training

Author DC first received training on helminth identification and enumeration from staff at the Mozambican National Institute of Health's Parasitology Lab in Maputo, Mozambique. Second, author DC received training in mini-FLOTAC and helminth identification and enumeration from the Kaplan Lab at the University of Georgia, which also serves as the U.S. Distributor for mini-FLOTAC.

All laboratory technicians were trained by DC in helminth identification and enumeration, except author TB who previously worked as a technician in a veterinary parasitology laboratory. Laboratory technicians were trained over a period of 2 to 4 weeks. Technicians were required to read the following references: 1) World Health Organization's " Bench Aid for the Diagnosis of Intestinal Parasites" (First and Second Editions); 2) CDC DPDx's "Diagnostic Procedures for Intestinal Parasites" (https://www.cdc.gov/dpdx/diagnosticprocedures/stool/morphcomp.html); 3) CDC DPDx's "Artifact Identification Sheet" (https://www.cdc.gov/dpdx/artifacts/index.html); 4) Donald L. Price's "Procedure Manual for the Diagnosis of Intestinal Parasites"; and 5) Ash and Orihel's "Human Parasitic Diseases: A Diagnostic Atlas." Then, technicians were trained for 1 day on using a microscope and practiced identifying ova that were fixed and mounted onto prepared slides (VWR, Radnor, PA). The study team acquired feces from dogs, cats, chickens, pigs, horses, and cows that contained a wide range of helminth ova (e.g., hookworm, *Ascaris*, *Trichuris*, *Toxocara*, *strongyloides*, and pinworm) and artifacts (e.g., pollen, undigested food,

and mite eggs). Technicians received a day of instruction on sodium nitrate solution preparation and the mini-FLOTAC method from either DC or TB, and continued a self-guided period of training with different stool samples for 2–3 weeks. Frozen human stool samples collected as part of the MapSan Trial (*1*) that contained *Ascaris* and *Trichuris* ova were used in training. As the final step in training lab technicians had to demonstrate the ability to enumerate ova within 25% of the count observed by DC. Finally, during the analysis phase of the study, technicians consulted DC and TB for help identifying ova if they were uncertain.

Combined Sensitivity

Mini-FLOTAC Sensitivity

We took a highly conservative approach to estimate the sensitivity of mini-FLOTAC. First, we consulted Cools *et al*. 2019 (*2*), which in Figure 2 reports a sensitivity of 21% for 0–49 eggs per gram and 82% for 50–149 eggs per gram. As these were reported as ranges, we assumed that these values best represented the sensitivity of the median value in the range, which were 25 and 100 respectively. We fit a linear regression line (slope = 0.0081) between these two values to interpolate the sensitivity for egg per gram values between 25 and 100. Assuming the sensitivity of stool with no ova is 0, we used the same methodology to interpolate individual sensitivities (slope = 0.0084) from 0 to 25 ova per gram. For 1 ovum per gram the calculation is as follows:

Eq 1. $Sensitivity_n = Sensitivity_{25} - \left(\frac{21\%}{25}\right) * (25 - n) = 21 - \left(\frac{21\%}{25}\right) * (25 - 1) = 21 - 20.16 = 0.84\%$

We also accounted for degradation of the ova in 10% formalin. In Appendix Figure S3 we found that egg counts reduced by 0.0049 $\log_{10}$ per day on average. The median number of days between sample collection and analysis was 28. This suggests that if a stool sample contained ova, the concentration would have decreased by 0.14 $\log_{10}$ from collection to analysis. We subtracted this value from the initial eggs per gram, and then used this value to calculate the sensitivity after considering egg degradation. For example, if 1 ova per gram was initially present in the stool, the concentration following preservation would be:

Eq 2. $Concentration_n = Concentration_0 - 0.14\, log_{10} = 0\, log_{10} - 0.14\, log_{10} = -0.86\, log_{10} = 10^{-0.14} = 0.73$ ova per gram

Then we re-calculated the $Sensitivity_n$ for the concentration adjusted to reflect egg degradation in 10% formalin. Replacing n in equation 1 with 0.73, instead of 1, we calculated the sensitivity of a single mini-FLOTAC test to be 0.61% for stool that contained 1 ovum initially upon defecation. However, we did the analysis in triplicate. We calculated the sensitivity of this triplicate analysis with equation 3, which reports the sensitivity of 1 egg per gram.

Eq 3. $Sensitivity_{triplicate,n} = 1 - \left(1 - Sensitivity_{single}\right)^3 = \; Sensitivity_{triplicate,1} = 1 - (1 - 0.61)^3 = 1.8\%$

We repeated this methodology to calculate the sensitivity of each integer value from one to a hundred.

qPCR and dPCR Sensitivity

We first quantified the number of gene copies in a hookworm ovum to determine the sensitivity of our molecular methods. We collected three fecal samples from canines infected with *Ancylostoma caninum* at a local animal hospital. Then we enumerated the number of eggs per gram by performing mini-FLOTAC in triplicate. Next, we extracted total nucleic acids from 100 mg of each stool sample in triplicate. Finally, we quantified gene copies of *Ancylostoma caninum* using digital PCR (QIAcuity 4, Qiagen, Hilden, and Germay). Dividing the number of gene copies by the number of ova per stool indicated a mean of 2,220 gene copies per ovum (IQR = 437, 3600).

Next, we considered the dilution during sample processing. The dilutions used would have required 5,500 gene copies and 825 gene copies, for qPCR and for dPCR respectively, of the target sequence present per gram of feces for one gene copy to be theoretically present in the respective PCR reaction. However, one gene copy is unlikely to consistently amplify in a PCR reaction. We accounted for this by analyzing replicates of low concentrations (e.g., 10^{-1}, 10°, 10^1, 10^2 copies per μL) of an engineered plasmid (*3*) to determine the 95% limit of detection (LOD) using the methods described in Stokdyk et al. 2016 (Apendix Figure 1) (*4*). We determined the concentration of the plasmid based on the mass of an individual plasmid and the total quantity of DNA provided by the manufacturer (GeneArt ThermoFisher Scientific,

Waltham, Massachusetts). With these methods we determined the 95% LOD for qPCR was 4.0 gene copies per uL template and for dPCR was 0.40 gene copies per uL template. In our dPCR reaction, we used four uL of template, which suggests the 95% LOD was 1.6 gene copies per reaction. However, we required three positive partitions for a sample to be considered positive following manual thresholding based on the performance of our negative controls (Appendix Table 3, Appendix Table 4). We accounted for this by substituting the calculated 95% LOD for dPCR – which was 1.6 gene copies per reaction – with 3.0 gene copies per reaction in our sensitivity calculation and allow for a more conservative estimate. Combining the estimated LOD with our dilutions, we determined there was a 95% chance of detecting hookworm DNA at concentrations of 21,896 gene copies per gram stool for qPCR and 2,475 for dPCR.

Similar to our methods for mini-FLOTAC, we also considered the potential decay of DNA in the Zn-PVA preservation buffer between sample collection and analysis. Using the canine feces described previously to quantify the number of gene copies of *Ancylostoma caninum* per ovum, we aliquoted these feces into different preservation buffers under different storage conditions and extracted nucleic acids over time (Appendix Figure 4). The results indicated a 0.033 $\log_{10}$ reduction in the concentration of hookworm DNA per day in ZnPVA at ambient conditions and a 0.015 $\log_{10}$ reduction at 4°C. We tracked the time between sample collection, receipt, and analysis. There was a median of 14 days at ambient conditions, which occurred before receipt at the lab, and a median of 15 days for storage at 4°C before analysis. This combined decay suggests the initial concentration of hookworm DNA would have decreased by 0.70 $\log_{10}$ from sample collection to analysis. Combining our 95% LODs with the estimated decay enables an estimate of what initial concentration would have been necessary to detect hookworm DNA using our methods. This is demonstrated in Equation 4.

Eq 4. $\log_{10} Gene\ Copies_{decay} = \log_{10} Gene\ Copies_{LOD} + 0.70$

The equation indicates that for qPCR 110,322 gene copies and for dPCR 12,470 gene copies would have needed to be present per gram of feces upon sample collection to have a 95% chance of positive detection. Given that there are 2,200 gene copies per hookworm ova, the methods provided a 95% chance of detecting 47.2 ova per gram via qPCR and 5.3 ova per gram via dPCR. The individual sensitivities are then calculated by Equation 5.

Eq 5a. $Sensitivity_{n,qPCR} = n * \frac{2,200}{110,322}$

Eq 5b. $Sensitivity_{n,dPCR} = n * \frac{2,200}{12,470}$

The combined sensitivity was calculated using equation 6.

Eq 6. $Sensitivity_{overall} = 1 - (1 - Sensitivity_{FLOTAC})(1 - Sensitivity_{qPCR})(1 - Sensitivity_{dPCR})$

Geospatial Map methodology

Geospatial maps were created using geographic information system GIS software (Esri ArcPro 2.8). Participant residential address locations with attributes related to well water and sanitation type were geocoded using ArcPro and Esri StreetMap Premium. Maps were cartographically designed to maintain participant privacy using heat maps to display general distribution rather than exact locations.

References

1. Knee J, Sumner T, Adriano Z, Anderson C, Bush F, Capone D, et al. Effects of an urban sanitation intervention on childhood enteric infection and diarrhea in Maputo, Mozambique: a controlled before-and-after trial. eLife. 2021;10:10. PubMed https://doi.org/10.7554/eLife.62278

2. Cools P, Vlaminck J, Albonico M, Ame S, Ayana M, José Antonio BP, et al. Diagnostic performance of a single and duplicate Kato-Katz, Mini-FLOTAC, FECPAKG2 and qPCR for the detection and quantification of soil-transmitted helminths in three endemic countries. PLoS Negl Trop Dis. 2019;13:e0007446. PubMed https://doi.org/10.1371/journal.pntd.0007446

3. Kodani M, Winchell JM. Engineered combined-positive-control template for real-time reverse transcription-PCR in multiple-pathogen-detection assays. J Clin Microbiol. 2012;50:1057–60. PubMed https://doi.org/10.1128/JCM.05987-11

4. Stokdyk JP, Firnstahl AD, Spencer SK, Burch TR, Borchardt MA. Determining the 95% limit of detection for waterborne pathogen analyses from primary concentration to qPCR. Water Res. 2016;96:105–13. PubMed https://doi.org/10.1016/j.watres.2016.03.026

5. Liu J, Gratz J, Amour C, Nshama R, Walongo T, Maro A, et al. Optimization of quantitative PCR methods for enteropathogen detection. PLoS One. 2016;11:e0158199. PubMed https://doi.org/10.1371/journal.pone.0158199

6. Rudko SP, Ruecker NJ, Ashbolt NJ, Neumann NF, Hanington PC. *Enterobius vermicularis* as a novel surrogate for the presence of helminth ova in tertiary wastewater treatment plants. Appl Environ Microbiol. 2017;83:e00547-17. PubMed https://doi.org/10.1128/AEM.00547-17

7. Köller T, Hahn A, Altangerel E, Verweij JJ, Landt O, Kann S, et al. Comparison of commercial and in-house real-time PCR platforms for 15 parasites and microsporidia in human stool samples without a gold standard. Acta Trop. 2020;207:105516. PubMed https://doi.org/10.1016/j.actatropica.2020.105516

8. Camacho-Sanchez M, Burraco P, Gomez-Mestre I, Leonard JA. Preservation of RNA and DNA from mammal samples under field conditions. Mol Ecol Resour. 2013;13:663–73. PubMed https://doi.org/10.1111/1755-0998.12108

9. Hill VR, Narayanan J, Gallen RR, Ferdinand KL, Cromeans T, Vinjé J. Development of a nucleic acid extraction procedure for simultaneous recovery of DNA and RNA from diverse microbes in water. Pathogens. 2015;4:335–54. PubMed https://doi.org/10.3390/pathogens4020335

Appendix Table 1. TAC performance of assay used in study conducted in Alabama, USA, December 2019–August 2022

Target	Target Gene	R^2	Efficiency	Reference
Ancylostoma duodenale	ITS-2	1.000	98%	(*5*)
Ascaris lumbricoides	ITS-1	1.000	95%	(*5*)
Enterobius vermicularis	5S	0.999	95%	(*6*)
Hymenolepsis nana	ITS-1	1.000	98%	(*7*)
Necator americanus	ITS-2	1.000	98%	(*5*)
Strongyloides stercolaris	Dispersed repetitive sequence	0.999	100%	(*5*)
Trichuris trichiura	18S rRNA	1.000	99%	(*5*)

Appendix Table 2. MIQE Checklist for TAC analysis of fecal samples from children enrolled in Lowndes, Perry, and Wilcox Counties in a study conducted in Alabama, USA, December 2019–August 2022

Item to check	Importance	Checklist
Experimental design		
Definition of experimental and control groups	E	There were no experimental or control groups.
Number within each group	E	We ran stool from 488 children on the custom TAC via RT-qPCR.
Sample		
Description	E	Children’s stool samples preserved in Zn-PVA
Processing procedure	E	Described in the methods section
Sample storage conditions and duration (especially for FFPE samples)	E	Described in the results section
Nucleic acid extraction		
Procedure and/or instrumentation	E	QIAamp 96 Virus QIAcube HT Kit on a QIAcube HT
Name of kit and details of any modifications	E	We mixed 150 mg of the stool ZnPVA mixture with 1 mL of Qiagen Buffer ASL in Precellys® SK38 bead beating tubes, vortexed to bead beat for five minutes, incubated at room temperature for 15 min, centrifuged at 14000 rpm for 2 min, and then transferred 200 uL of supernatant to the QIAcube to proceed with extraction using the manufacturer’s default procedure for the QIAamp 96 Virus QIAcube HT Kit.
Details of DNase or RNase treatment	E	None
Contamination assessment (DNA or RNA)	E	We included one negative extraction control on each day of extractions.

Item to check	Importance	Checklist
Nucleic acid quantification	E	We measured nucleic acids using qubit on a subset of samples
Instrument and method	E	Qubit 4 Fluoremeter, 1X dsDNA High Sensitivity
RNA integrity method/instrument	E	Not performed
Inhibition testing (Cq dilutions, spike or other)	E	We spiked in a DNA and RNA control into each extraction (see methods section).
Reverse transcription		
Complete reaction conditions	E	One-step reverse transcription
Amount of RNA and reaction volume	E	We combined 40 µL of template with 60 µL of AgPath-ID One-Step RT-PCR Reagents. The reaction volume was 1.5 µL.
Priming oligonucleotide (if using GSP) and concentration	E	Applied Biosystems, AgPath-ID One-Step RT-PCR Reagents Catalog number: 4387391
Reverse transcription and concentration	E	ArrayScript Reverse transcription
Temperature and time	E	45°C for 20 min
qPCR target information		
If multiplex, efficiency and LOD of each assay.	E	Appendix Table 1
In silico specificity screen (BLAST, etc)	E	We BLASTed all assays to confirm specificity before ordering the custom TAC.
qPCR oligonucleotides		
Primer sequences	E	Citations for primer and probe sequences are listed in Appendix Table 1.
Location and identity of any modifications	E	None
qPCR protocol		
Complete reaction conditions	E	45°C for 20 min and 95°C for 10 min, followed by 45 cycles of 95°C for 15 s and 60°C for 1 min
Reaction volume and amount of cDNA/DNA	E	100µL reactions were prepared, containing 60uL of mastermix and 40µL of template. This corresponds S12 to 0.6µL of template and 0.9µL of mastermix per reaction well.
Primer, (probe), Mg++ and dNTP concentrations	E	All assays contained the same concentrations of primers (900 nmol/L) and probe (250 nmol/L). The Mg2+ and dNTP concentrations are not listed in the in the User Guide.
Polymerase identity and concentration	E	AmpliTaq Gold polymerase
Buffer/kit identity and manufacturer	E	AgPath-ID One-Step RT-PCR Reagents
Additives (SYBR Green I, DMSO, etc.)	E	None
Complete thermocycling parameters	E	45°C for 20 min and 95°C for 10 min, followed by 45 cycles of 95°C for 15 s and 60°C for 1 min
Manufacturer of qPCR instrument	E	ThermoFisher Scientific
qPCR validation		
Specificity (gel, sequence, melt, or digest)	E	See references listed in Appendix Table 1.
PCR efficiency calculated from slope	E	See Appendix Table 1
r2 of standard curve	E	See Appendix Table 1
Evidence for limit of detection	E	See Appendix Figure 1
If multiplex, efficiency and LOD of each assay.	E	All assays were singleplex
Data analysis		
qPCR analysis program (source, version)	E	QuantStudio Real-Time PCR Software V1.2 CDC
Cq method determination	E	Manual thresholding
Results of NTCs	E	For PCR run on the TAC platform, we did not observe contamination among extraction negative controls (n = 19) or PCR negative controls (n = 2), and our PCR positive controls (n = 30) exhibited the expected amplification for all targets. For dPCR we did not observe contamination among NTCs (n = 16) and positive controls exhibited positive partitions (n = 14).
Description of normalization method	E	Mass of stool extracted from (150 mg)
Number and stage (RT or qPCR) of technical replicates	E	Explained in the corresponding publication Capone et al.

Appendix Table 3. dMIQE Checklist for dPCR assay to detect *N. americanus* in fecal samples from children enrolled in Lowndes and Wilcox County in a study conducted in Alabama, USA, December 2019–August 2022

Item to check	Provided, Y/N	Comment
1. Specimen		
Detailed description of specimen type and numbers	Y	We ran Zn-PVA preserved stool from 265 children
Sampling procedure (including time to storage)	Y	Described in methods section
Sample aliquotation, storage conditions and duration	Y	Described in results section
2. Nucleic acid extraction		
Description of extraction method including amount of sample processed	Y	We mixed 150 mg of the stool ZnPVA mixture with 1 mL of Qiagen Buffer ASL in Precellys® SK38 bead beating tubes, vortexed to bead beat for five minutes, incubated at room temperature for 15 min, centrifuged at 14000 rpm for 2 min, and then transferred 200 uL of supernatant to the QIAcube to proceed with extraction using the manufacturer's default procedure for the QIAamp 96 Virus QIAcube HT Kit.
Number of extraction replicates	N	None in dPCR
Extraction blanks included	N	N/A
3. Nucleic acid assessment and storage		
Method to evaluate quality of nucleic acids	N	Not performed
Method to evaluate quantity of nucleic acids (including molecular weight and calculations when using mass)	Y	We measured nucleic acids using qubit on a subset of samples
Storage conditions: temperature, concentration, duration, buffer, aliquots	Y	Described in the results section
Clear description of dilution steps used to prepare working DNA solution	Y	None
4. Nucleic acid modification		
Template modification (digestion, sonication, pre-amplification, bisulphite etc.)	N/A	None performed
Details of repurification following modification if performed	N/A	None performed
5. Reverse transcription	N/A	None performed
6. dPCR oligonucleotides design and target information		
Sequence accession number or official gene symbol	Y	MH665842.1
Method (software) used for design and *in silico* verification	Y	NCBI BLAST
Location of amplicon	Y	453 to 474
Amplicon length	Y	102
Primer and probe sequences (or amplicon context sequence)**	Y	5′ ->3′<br>Fwd: CTGTTTGTCGAACGGTACTTGC<br>Rev: ATAACAGCGTGCACATGTTGC<br>Probe: 56FAM/CTGTACTACGCATTGTATAC/3MGB-NFQ
Manufacturer of oligonucleotides	Y	(IDT, Coralville, IA)
7. dPCR protocol		
Manufacturer of dPCR instrument and instrument model	Y	QIAGEN QIAcuity Four machine (Qiagen, Hilden, Germany)
Buffer/kit manufacturer	Y	QIAcuity Probe PCR Kit (5 ml) (Qiagen, Hilden, Germany)<br>Cat. No. / ID: 250102
Primer and probe concentration	Y	Probe: 400nM, Primers: 800nM
Pre-reaction volume and composition	Y	2µL template
Template treatment (initial heating or chemical denaturation)	N/A	None
Polymerase identity and concentration, Mg++ and dNTP concentrations***	N/A	Proprietary (QIAcuity Probe PCR Kit) (Qiagen, Hilden, Germany)
Complete thermocycling parameters	Y	1 × 95°C for 2 min<br>40 cycles x 95°C for 15 s, 50°C for 60 s
8. Assay validation		
Details of optimization performed	Y	This assay was optimized for our QIAcuity Four dPCR platform by systematically titrating probe and primer concentrations at varying annealing temperatures, aimed at maximizing separation between positive and negative bands and minimizing background noise (i.e., rain). First, primer concentrations of 400, 800, and 1600nM were tested at annealing temperatures of 50°C, 55°C, and 60°C. Next,

Item to check	Provided, Y/N	Comment
		probe concentrations of 200, 400, and 800nM were assessed. The primer, probe, and temperature combination with the greatest reaction efficiency was then selected.
Analytical sensitivity/LoD and how this was evaluated	Y	See Appendix Figure 1
9. Data analysis		
Comprehensive details negative and positive of controls (whether applied for QC or for estimation of error)		See “Controls” in “Results”
Partition classification method (thresholding)	Y	Threshold manually set to 100 RFU
Examples of positive and negative experimental results (including fluorescence plots in supplemental material)	N	All samples and NTCs ran on the QIAcuity Nanoplate 26k 24-well returned negative experimental results. We observed positive band hits for the positive control.
Description of technical replication	Y	6 samples were randomly selected to be run in duplicate, with all duplicates returning the same negative experimental results as the original samples. Duplicates were run using the same methodology from the same sample aliquots as the originals indicating reproducibility.
Plate type	Y	QIAcuity Nanoplate 26k 24-well (Qiagen, Hilden, Germany)
dPCR analysis program (source, version)	Y	QIAcuity software suite version 1.2 (Qiagen, Hilden, Germany).
Description of normalization method	Y	Mass of stool extracted from (150 mg)

Appendix Table 4. *N. Americanus* dPCR Data Summary of fecal samples from children enrolled in Lowndes and Wilcox Counties in a study conducted in Alabama, USA, December 2019–August 2022

Positive Controls	Value
Total Number Assayed	14
Average valid partitions per sample	24,433
Average positive partitions per sample	7,725
Samples with ≥3 partitions positive	14/14
Negative Controls	
Total Number Assayed	16
Average valid partitions per sample	25,412
Average positive partitions per sample*	0.25
Samples with ≥3 partitions positive	0/16
Stool Samples	
Total Number Assayed	265
Average valid partitions per sample	25,415
Average positive partitions per sample *	0.06
Samples with ≥3 partitions positive	0/265
Number of duplicate samples analyzed	30

*Among our 16, no template controls two had one positive partition and one had two positive partitions. Based on this data, and best practice with digital PCR, we only considered samples positive if three or more partitions were above the line of manual thresholding (Appendix Figure 2).

Appendix Table 5. Decay constants for *Ancylostoma caninum* DNA in different preservation buffers

Target	Preservative	log10 decay of DNA per day
Ancylostoma caninum	Zn PVA (4°C)	−0.0147
	Zn PVA (20°C)	−0.0331
	UNEX	−0.0079
	TotalFix	−0.0267
	NAP	−0.0010
	70% Ethanol	−0.0228

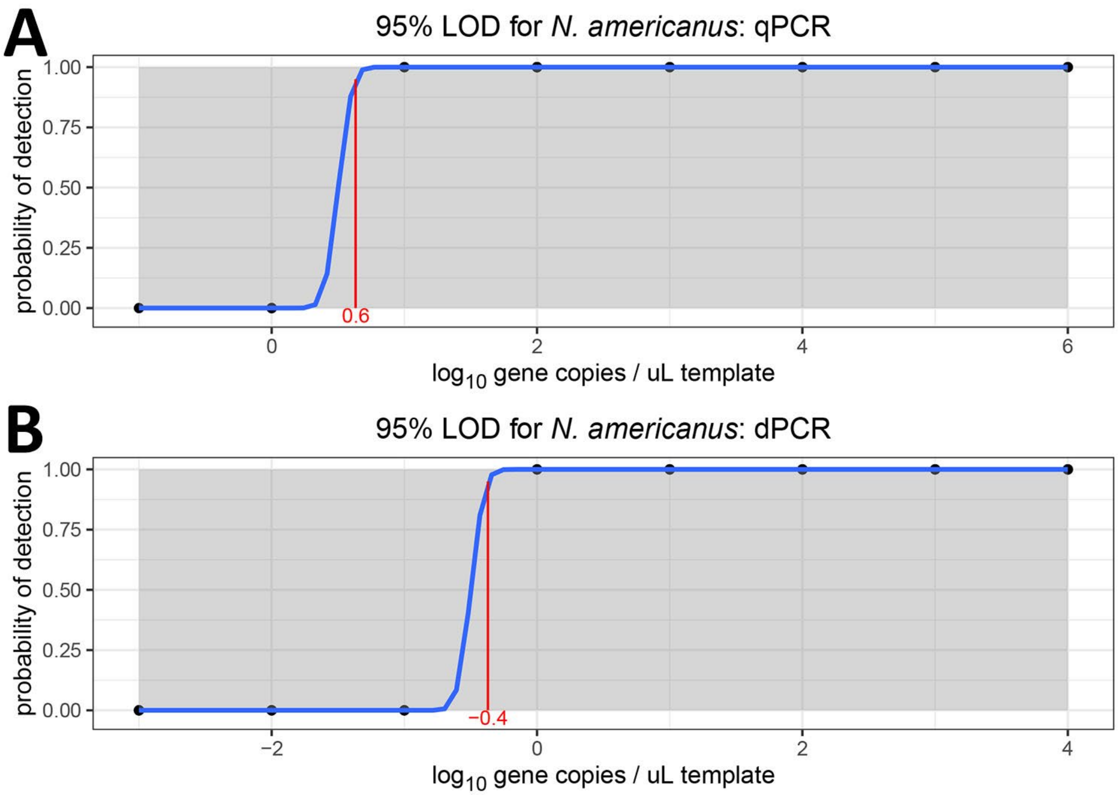

Appendix

Figure 1. 95% Limit of detection for *N. americanus*: qPCR for assay used in study conducted in Alabama, USA, December 2019–August 2022.

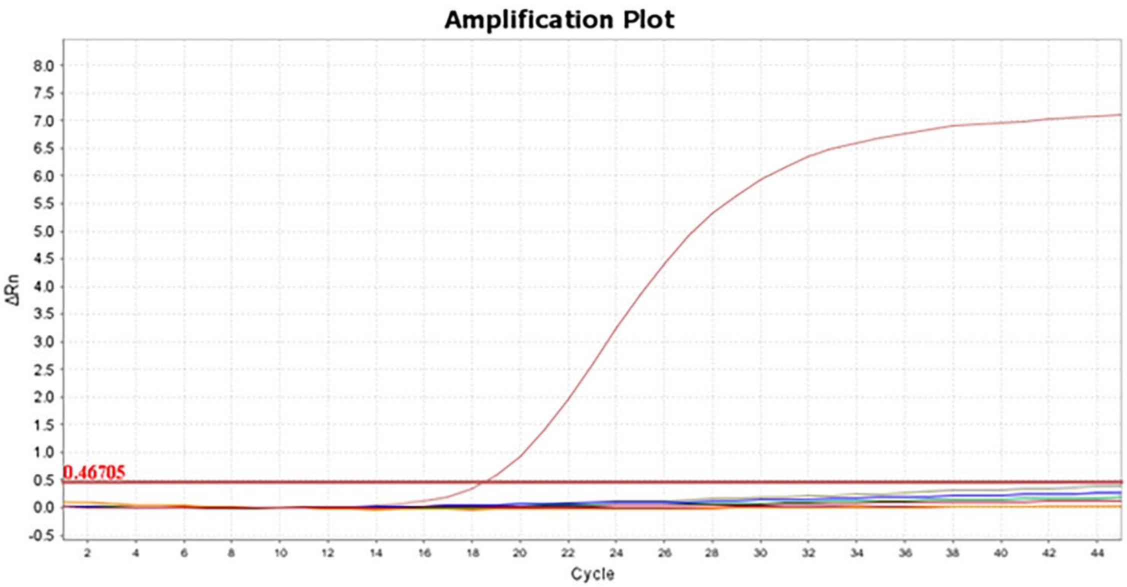

Appendix Figure 2. Amplification and Multicomponent Plots used to determine quantification cycle for TAC analysis used in study conducted in Alabama, USA, December 2019–August 2022. The positive control amplified but the negative control and samples do not.

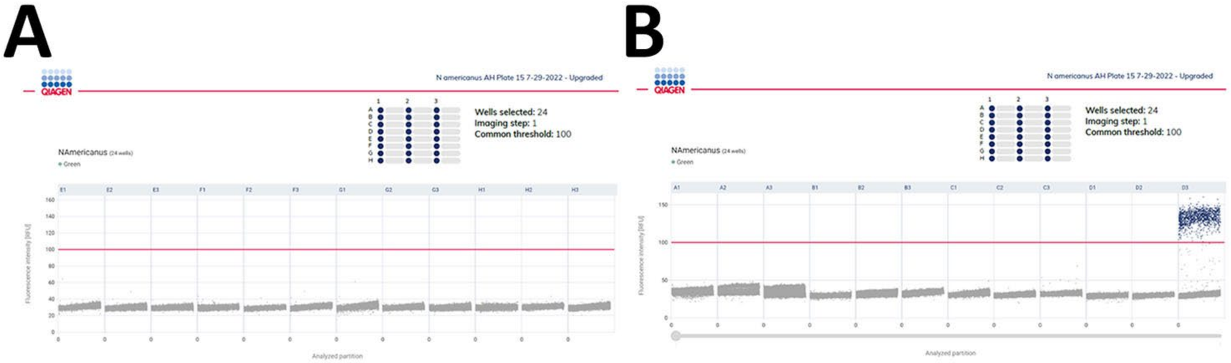

Appendix Figure 3. Screenshots from a nanoplate run for *Necator Americanus* by dPCR assay used in study conducted in Alabama, USA, December 2019–August 2022. All samples were negative except the positive control.

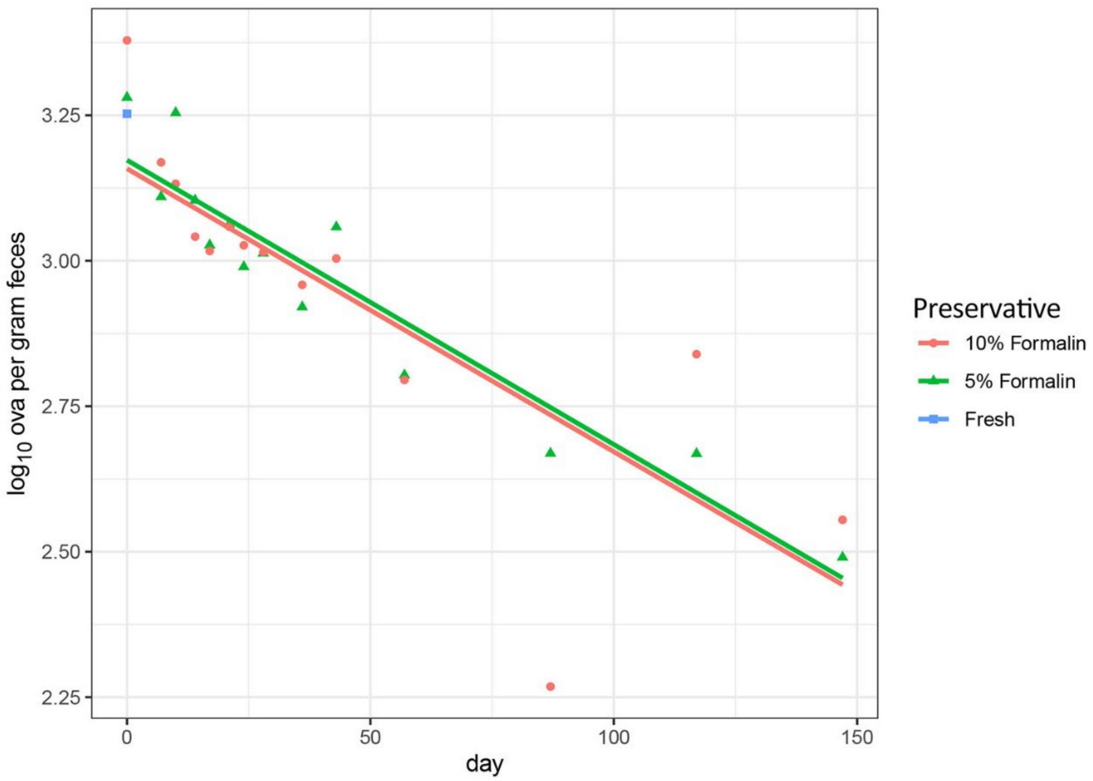

Appendix Figure 4. Recovery Experiment of *Ancylostoma caninum* from canine feces in 10% and 5% formalin over time. We received canine feces containing *Ancylostoma caninum* from the Kaplan Lab in the College of Veterinary Medicine at the University of Georgia. Upon receipt, we aliquoted and homogenized a portion of the stool 1:1 into 10% Formalin and into 5% Formalin. Then we enumerated the fresh (2 g) and preserved stool (4 g of the Formalin-Stool mixture) using mini-FLOTAC in triplicate. The

preserved stool was stored at room temperature and hookworm ova were enumerated weekly, and then monthly, over a period of 5 months. We observed a loss of 0.0049 log10 ova per day in both 5% and 10% Formalin.

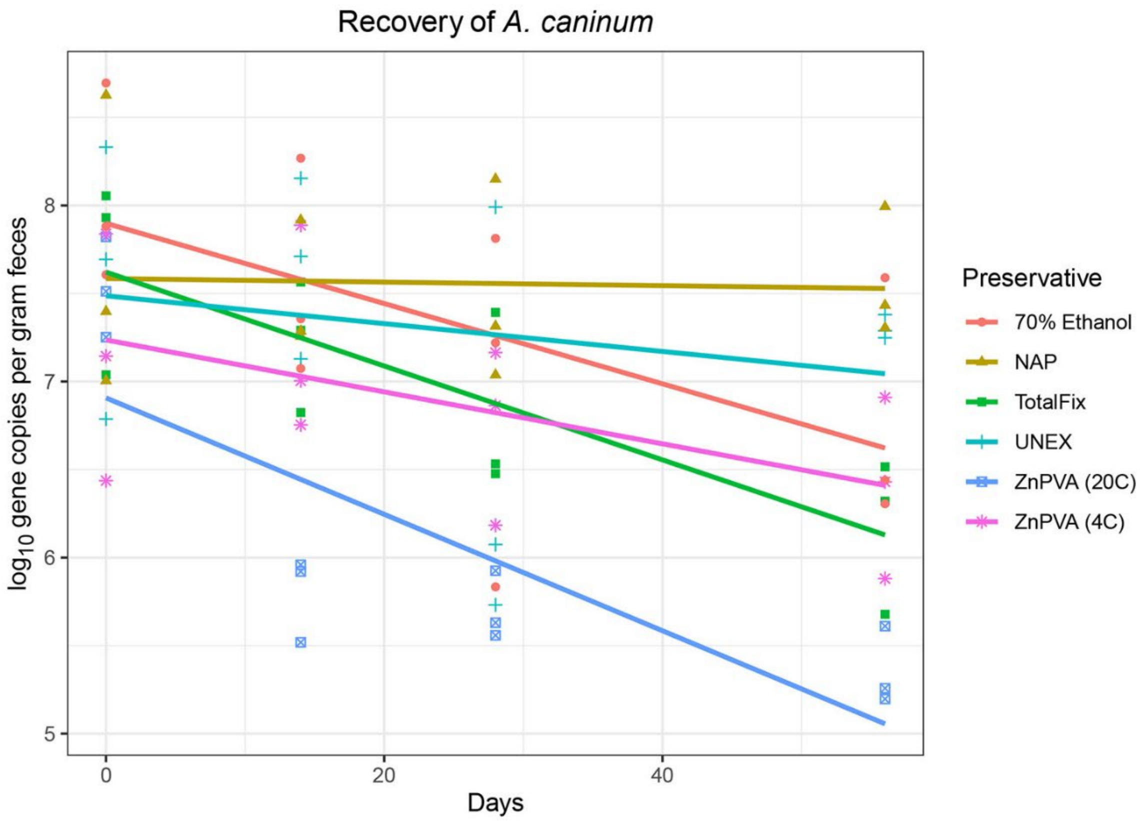

Appendix Figure 5. Nucleic Acid Recovery Experiment using *Ancylostoma caninum* comparing different fecal sample preservatives. We collected feces from three dogs at an animal hospital in rural North Carolina, enumerated hookworm ova using mini-FLOTAC and confirmed the species was *Ancylostoma caninum* using dPCR. We combined aliquots of the feces 1:1 with five different preservatives: ZnPVA at 4°C, ZnPVA at ambient (i.e., 20°C), 70% ethanol, TotalFix, homemade Nucleic Acid Preservation Buffer (NAP, Camacho-Sanchez *et al.* 2013) (*8*), and homemade Universal Extraction Buffer (UNEX, Hill *et al.* 2015) (*9*). We extracted nucleic acids from each aliquot on the day of sample preparation (i.e., Day 0), as well as Day 14, 28, and 56. We observed that our recovery of nucleic acids from *Ancylostoma caninum* decreased by 0.0331 log10 per day in ZnPVA at ambient conditions and by 0.0147 log10 per day in ZnPVA at 4°C. The best-performing preservation buffer was NAP.
